# Langevin equations from experimental data: The case of rotational diffusion in granular media

**DOI:** 10.1371/journal.pone.0212135

**Published:** 2019-02-22

**Authors:** Marco Baldovin, Andrea Puglisi, Angelo Vulpiani

**Affiliations:** 1 Dipartimento di Fisica, Sapienza Università di Roma, p.le A. Moro 2, 00185 Roma, Italy; 2 CNR-ISC and Dipartimento di Fisica, Sapienza Università di Roma, p.le A. Moro 2, 00185 Roma, Italy; 3 Centro Linceo Interdisciplinare “B. Segre”, Accademia dei Lincei, Rome, Italy; International Prevention Research Institute, FRANCE

## Abstract

A model has two main aims: predicting the behavior of a physical system and understanding its nature, that is how it works, at some desired level of abstraction. A promising recent approach to model building consists in *deriving* a Langevin-type stochastic equation from a time series of empirical data. Even if the protocol is based upon the introduction of drift and diffusion terms in stochastic differential equations, its implementation involves subtle conceptual problems and, most importantly, requires some prior theoretical knowledge about the system. Here we apply this approach to the data obtained in a rotational granular diffusion experiment, showing the power of this method and the theoretical issues behind its limits. A crucial point emerged in the dense liquid regime, where the data reveal a complex multiscale scenario with at least one fast and one slow variable. Identifying the latter is a major problem within the Langevin derivation procedure and led us to introduce innovative ideas for its solution.

## Introduction

The Langevin equation is surely one of the pillars of non equilibrium statistical mechanics [1]. Such a stochastic process has been introduced more than one century ago by Langevin in his seminal paper on the Brownian motion of colloidal particles in a fluid. In a nutshell the basic idea is the following: in a system with some slow variables it is possible to model the dynamics of these observables with an effective stochastic equation containing a systematic drift and a noisy term.

The study of Brownian motion played a crucial role to establish, in a conclusive way, the physical validity of the atomic hypothesis [2]. In his celebrated work Langevin had been able to write the Brownian evolution law with a deep intuition and a clever combination of macroscopic and microscopic ingredients (namely the Stokes law and energy equipartition). This work had been one of the starting points of the mathematical theory of continuous stochastic processes, namely stochastic differential equations, which are basically a generalisation of the Langevin equation [3–5]. In the following we will use “Langevin equation” with the loose meaning of stochastic differential equation, an identification broadly accepted in the literature.

Unfortunately a stochastic differential equation can be derived from a microscopic description with a systematic approach just in few cases. One important example is the diffusion of a big heavy intruder in a diluted gas of light particles: the kinetic theory allows to determine the stochastic differential equation ruling the evolution of the velocity of the heavy particle in terms of the microscopic parameters [6].

Another system where it is possible to build the Langevin equation with an analytical approach is a large harmonic chain containing *N* particles, one of which much heavier than the others, in the limit *N* ≫ 1 [7].

As far as we know, the origin of the (few) successes in the derivation of a stochastic differential equation with a non phenomenological approach can be always related to the high dilution of the system or to its linear character.

On the other hand, typically, it is necessary to adopt a more pragmatic attitude combining mathematics (if possible), intuition, suggestions from the data, and a preliminary understanding of the system under investigation. The present paper aims at applying a recently developed method to derive a Langevin equation from a time series of data [8, 9]. This method relies upon the basic textbook definitions of drift and diffusion coefficients of a stochastic differential equation, but in its applications it has been refined under many aspects, which are not only technical but also conceptual. Some difficulties are related to the coexistence of two properties of the Langevin equation, i.e. continuity and Markovianity. The most relevant conceptual issue is that of determining the proper variables for a complete Markovian description, which is not trivial when the available data are constituted by a single variable time series: in the theory of dynamical systems the so-called embedding theorem (due to Takens) provides a procedure—in principle of general validity—to reconstruct the correct phase space [10], once one assumes that the system is deterministic. Unfortunately such a procedure in many specific cases, including the one discussed here, is not useful and must be replaced by new ideas. Methods based on a stochastic approach are widely used in order to model and analyse complex features in a general setting, e.g. 1/*f* noise, see for instance [11–13]; our aim here is to find effective Markov models, with quantitative determination of parameters, for a specific system.

The present paper is organized as follows. In the Materials and Methods section we first discuss the procedure of Langevin derivation from data, with its practical and conceptual difficulties and, second, we revise the experimental setup (rotational diffusion in diluted and dense granular gases) and some previous phenomenological models adopted to understand such experiment. In the Results section we apply the method to the data, in particular in a dilute and a dense case, which are extremely different cases. In the Conclusion section the reader can find general and conclusive remarks.

## Materials and methods

### Langevin equation from data

Let us present a direct approach to build a Langevin equation from data. As far as we know, in spite of its (relatively) easy use, there has been just few attempts in such a direction. Likely it is due to the fact that, although the method can appear quite obvious and easy at a first glance, there are some rather severe difficulties both at conceptual and technical level.

Let us assume that we know that the slow “good” variables are the component of a vector **X**(*t*) ∈ *R*^*N*^. In the following we will see that this is a rather subtle point, and the choice of the proper **X** is not easy at all. Let us also suppose that we have measured, e.g. in an experiment or a simulation, a long time series of these variables, {**X**(*t*)}. With these assumptions, one can determine the *N* Langevin equations (*n* ∈ [1, *N*])

$$\begin{array}{c} \frac{dX_{n}}{dt}=F_{n}\left(X\right)+\sqrt{2D_{n}\left(\text{X}\right)}\eta_{n}, \end{array}$$ (1)

where *η*_*n*_(*t*) are white noises i.e. Gaussian processes with 〈*η*_*n*_(*t*)〉 = 0 and 〈*η*_*n*_(*t*)*η*_*n*′_(*t*′)〉 = *δ*_*nn*′_*δ*(*t* − *t*′), following the definitions found in textbooks on stochastic processes [3–5], that is in terms of the statistical features of

$$\Delta X_{n}\left(\Delta t\right)=X_{n}\left(t+\Delta t\right)-X_{n}\left(t\right).$$

In fact the drifts and diffusion coefficients are given by the following formula:

$$\begin{array}{c} \begin{array}{cc} F_{n}\left(\text{X}\right) & =\lim_{\Delta t\to 0}\frac{1}{\Delta t}\langle \Delta X_{n}\left(\Delta t\right)|\text{X}\left(t\right)=\text{X}\rangle \\ D_{n}\left(\text{X}\right) & =\lim_{\Delta t\to 0}\frac{1}{2\Delta t}\langle {\left(\Delta X_{n}\left(\Delta t\right)-F_{n}\left(\text{X}\right)\Delta t\right)}^{2}|\text{X}\left(t\right)=\text{X}\rangle \end{array} \end{array}$$ (2)

It is easy to realize, see Ref. [9], that the limit Δ*t* → 0 must be considered in a proper physical sense, i.e. smaller than typical time, but not too small. Sometimes the term “Langevin equation” is used with different meanings, here it indicates Eq (1) where **F** is not necessary linear and the noise may be multiplicative, i.e. *D* can depend upon **X**.

The practical procedure to extract the {*F*_*n*_} and {*D*_*n*_} from data is not trivial; however it is not the most difficult problem one has to face: the main conceptual trouble is indeed given by the absence a general method for choosing the “right” variables, an aspect that is too often overlooked. For instance, Onsager and Machlup are explicit in raising the question [14]:

How do you know you have enough variables, for [the system] to be Markovian?

Similarly, Shang-Keng Ma expresses a caveat of central importance [15]:

The hidden worry of thermodynamics is: we do not know how many coordinates or forces are necessary to completely specify an equilibrium state.

Usually the “proper variables” are unknown and in the building of the model one can use the time series of just one observable {*U*(*t*)}, or a few ones. Such problem is quite similar to the phase space reconstruction in dynamical systems. There are no automatic protocols for this choice, and typically to obtain some good results it is necessary to possess the expertise and/or intuition about the problem under investigation. For a general discussion on the difficulties to build models from data, see [16].

Sometimes mathematics can help to anticipate that a certain set of variables is not adequate as Markovian model. For instance, see Ref. [9], in the case of a single scalar variable, the shape of the correlation function may be already sufficient to exclude that such variable is an equilibrium Markov process, so that it becomes necessary to look for a new set of variables.

### Experimental setup and phenomenological models

Granular materials apparently share many properties with condensed “molecular” matter [17, 18], but such similitudes hide a crucial difference: grains, being macroscopic, dissipate energy through friction (in enduring contacts or rapid collisions). For this reason equilibrium statistical physics may only suggest qualitative ideas for fluidized steady states and dramatically fails in the extreme case of static or quasi-static regimes. The liquid state of granular matter, which is in the middle between fast “granular gases” and slow “granular glasses”, feels stronger the need for a coherent theoretical framework: continuum descriptions for dense flows lack first-principle constitutive relations [19, 20], so that transport coefficients can only be measured in molecular dynamics simulations [21], while kinetic theories (e.g. mode-coupling) must be carefully adapted to take into account some fundamental peculiarities, such as dissipation and inertia [22]. An important insight is provided by experiments, where such a liquid state is obtained through some mild shaking of the container [23–25]. In the setup described below our focus is on regimes where the longest relaxation time is reasonably smaller than the total experimental time, so that the system can be said to be in a *steady state*. In a word we are not interested, here, in the solid or glassy states [26–28].

**Fig 1:**
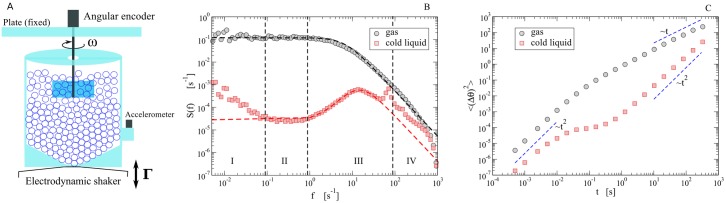
Experimental results. A: Sketch of the experiment reported in [25]. B: Experimental data of the VPDS for the gas case and the “cold liquid” case, together with predictions (dashed lines) from the incomplete model, Eq (5). C: Experimental data of the MSD for both cases, together with dashed lines useful as guides for the eye.

A recent experimental study [25] has offered a new picture for dense granular flows in a wide range of time-scales, from 10^−3^ s up to 10^3^ s and more, revealing an unexpectedly rich scenario. In the experimental setup, sketched in Fig 1A the “impurity” was constituted by an immersed blade who could rotate around a fixed vertical axis under the kicks from the grain of a vibrofluidized granular medium. The dynamics of the angular velocity *ω*(*t*) of the blade and its absolute angular position $\theta \left(t\right)=\int_{0}^{t}ds\omega \left(s\right)$, was studied in different regimes of density and intensity of vibration. In Fig 1B, the velocity power density spectrum (VPDS),

$$\begin{array}{c} S\left(f\right)=\frac{1}{2\pi t_{TOT}}{\left|\int_{0}^{t_{TOT}}\omega \left(t\right)e^{i(2\pi f)t}dt\right|}^{2}, \end{array}$$ (3)

is presented and its salient features are highlighted in two opposite limits, which are the gas and the cold liquid. We remind that the VPDS is the Fourier transform of the velocity autocorrelation function and that its *f* → 0^+^ limit is the self-diffusion coefficient, i.e. *D*_∞_ = *π* lim_*f*→0^+^_ *S*(*f*). We also recall that relations exist, under certain approximations, between the VPDS and the intermediate scattering function which—in liquids—is typically accessed through neutron scattering experiments [29].

In the gas limit (low packing fraction and high energy per grain) the probe velocity autocorrelation is close to a simple exponential decay $∼e^{-t/\tau_{gas}}$, ruled by a single relaxation time *τ*_*gas*_: in this limit the VPDS takes the form of a Lorentzian

$$\begin{array}{c} S\left(f\right)=\frac{T}{\pi \gamma }\frac{1}{1+{\left(2\pi If/\gamma \right)}^{2}}. \end{array}$$ (4)

In the—roughly speaking—opposite limit, that of a “cold liquid” (high packing fraction ≳ 30 – 35% and low energy per grain), the observed VPDS strongly deviates from the Lorentzian. Ignoring a mechanical resonance due to the mounting plate at ∼10^2^ *Hz*, it displays four different regions: at high frequency (region IV) it decays with a negative power law equal or smaller than 2; in region III it shows a smooth parabolic maximum (centered near ∼10*Hz*), reminiscent of a harmonic confinement (“cage”) typical of molecular and granular liquids [22, 30–32]; in region II it stabilizes on a short plateau, which suggests a loss of memory (as in the plateau of the Lorentzian which marks the onset of normal diffusion); finally region I, perhaps the most surprising one, shows a diverging *S*(*f*) for *f* → 0^+^, signaling a problem with the finiteness of the self-diffusion coefficient *D*_∞_. A few longer experiments (12 hours) were conducted, showing a slow crossover toward a new higher plateau at very low frequencies. The study of the mean squared displacement (MSD), see Fig 1C confirmed that the four regions of the cold liquid case correspond, respectively, to short-time ballistic (free) motion (IV), dynamical arrest due to caging (III), later relaxation of the cage (II) and “final” superdiffusive behavior (I), very rarely observed in previous works on granular systems [27, 28, 33, 34]. A universal scenario for anomalous diffusion is lacking [35], but certainly it is the signal of an enduring memory. A family of phenomenological models for anomalous diffusion includes fractional Fokker-Planck equations [36], where an immediate physical interpretation is not always at hand, or phenomenological continuous time random walk model for the *velocity* [37], with a power-law-decaying distribution of persistency times (see Supplemental Materials of [25]). Interestingly, simpler models—e.g. *linear* Langevin equations—can offer an even more complete insight in the many observed phenomena.

In [25] a first model was proposed to account for the caging phenomenon, i.e. regions II-III-IV of the VPDS in the cold liquid limit, inspired by the Itinerant Oscillator model for molecular liquids [38–40]; it is described by the following stochastic equations of motion:

$$\begin{array}{c} \begin{array}{cc} I\dot{\omega }\left(t\right) & =-\gamma \omega \left(t\right)-k\left[\theta \left(t\right)-\theta_{0}\left(t\right)\right]+\sqrt{2\gamma T}\eta \left(t\right)\\ \dot{\theta }\left(t\right) & =\omega (t)\\ {\dot{\theta }}_{0}\left(t\right) & =\sqrt{2D_{0}}\eta_{0}\left(t\right) \end{array} \end{array}$$ (5)

where *η*(*t*) and *η*_0_(*t*) are independent white normal Gaussian noises (unitary variance) and the angles *θ*(*t*) and *θ*_0_(*t*) are considered not bounded. The model represents the diffusion of a particle in a harmonic potential with “stiffness” *k* and unfixed minimum located at *θ*_0_(*t*), under the effect of a thermal bath at temperature *T* and relaxation time *I*/*γ*. The harmonic potential, representing the cage created by the confining effect of the dense granular host fluid, is not fixed but moves, as *θ*_0_(*t*) behaves as Brownian motion with diffusivity *D*_0_. Motivation for this model is twofold: 1) it reproduces the main features of the VPDS, i.e. short time fast relaxation (region IV), an elastic resonance at intermediate times (region III) and a plateau revealing loss of memory at larger times (region II); 2) in the dilute limit (when *k* → 0) there are analytical arguments for it [6, 41], 3) at intermediate densities a series of studies showed that memory effects (coming from correlated collisions) are well described by a similar coupling with an additional degree of freedom characterized by slower relaxation time-scales [42]. The VPDS of the above model is fully calculated in [25], fairly reproducing the VPDS in regions II-IV (see dashed lines in Fig 1B) in which *k*/*I* ∼ (2*π* ⋅ 10)^2^ ∼ 4 ⋅ 10^3^ *Hz*^2^.

In order to overcome the strong disagreement between the previous model and observations in region I (super-diffusion), a new model was introduced in [43]:

$$\begin{array}{c} \begin{array}{cc} I\dot{\omega }\left(t\right) & =-\gamma \omega \left(t\right)-k\left[\theta \left(t\right)-\theta_{0}\left(t\right)\right]+\sqrt{2\gamma T}\eta \left(t\right)\\ I_{0}{\dot{\omega }}_{0}\left(t\right) & =-\gamma_{0}\omega_{0}\left(t\right)+k\left[\theta \left(t\right)-\theta_{0}\left(t\right)\right]+\sqrt{2\gamma_{0}T_{0}}\eta_{0}\left(t\right)\\ \dot{\theta }\left(t\right) & =\omega (t)\\ {\dot{\theta }}_{0}\left(t\right) & =\omega_{0}\left(t\right) \end{array} \end{array}$$ (6)

In Eq (6) the angular velocity of the probe feels two different forces both related to collisions: one part is without memory and is described by the −*γω* + *η*(*t*) contribution, the second part takes the form −*k*[*θ*(*t*) − *θ*_0_(*t*)] and therefore depends upon the past history of *ω*(*t*) and *ω*_0_(*t*). As before *θ*_0_(*t*) should be viewed as a collective degree of freedom representing the preferential point of the blade with respect to some granular *cage*. The *cage* slowly changes its configuration and favors the blade’s drift at later times. This model however replaced the overdamped dynamics of *θ*_0_(*t*) in Eq (5), introducing the crucial effect of cage inertia *I*_0_. For the sake of symmetry a reciprocal effect of the blade upon the granular material was included, an ingredient which is likely to be negligible in view of the large value of *I*_0_. In [43] the introduction of cage inertia was motivated only by analogy: it was reasonable, when looking for an ingredient reproducing almost ballistic superdiffusion, to imagine that the cage (which, over long time-scales, dominates also the probe’s dynamics) is doing long ballistic drifts sustained by its large inertia. Discreteness and finiteness of the granular material, which in the experiments is made of a few thousands grains, makes random but persistent (also called “secular” [44]) drifts possible.

The linearity of the model allowed to solve it analytically, with a closed formula for many statistical properties, for instance the VPDS and, semi-analytically, also for the MSD. After some controlled procedure of fitting for the many parameters it was possible to find a perfect reproduction of the full dynamics in regions I-IV, including super-diffusion at long times, with a value of *I*_0_ of the same order of the moment of inertia of the granular medium surrounding the probe in the experiment [43].

## Results

In the previous section we have discussed how experimental data presented in [25] can be described by simplified stochastic models, whose parameters can be found by fitting dynamical observables as the VPDS or the MSD. In the following we will face the problem from a different, complementary, point of view: instead of checking the accordance of the measurements with a predeterminate theoretical model (based on physical arguments), we will try to get important information on the model itself directly from data, by enforcing the extrapolation protocol discussed above.

If the studied variable (namely, the angular velocity of the probe as a function of time) is a continuous Markov process, the procedure should be able to “automatically” find the best functional form for the corresponding Langevin Equation (provided that the sampling frequency is high enough). In the following we will examine a case, the gas limit, in which this scheme can be applied quite straightforwardly; in the cold-liquid limit, on the contrary, some additional considerations from physics will be needed—and not always sufficient—in order to get a satisfactory description.

### Gas limit

Let us consider first a “dilute gas” case: the container is filled with 350 grains, corresponding to a packing fraction of *ϕ* = 5%; the shaking intensity is $\Gamma =\ddot{z}/g=39.8$, where $\ddot{z}$ stands for the vertical acceleration and *g* = 9.81*m*/*s*^2^ is the gravitational acceleration. The measuring set-up records the angular position *θ*(*t*) of the blade with a sampling rate of *f*_*s*_ = 2000*Hz*, so that we can compute the angular velocity $\omega \left(t\right)=\dot{\theta }\left(t\right)$ with a temporal resolution of Δ*t*_*min*_ = 1/*f* = 0.5*ms*. Analyzing a long time series (1 hour) of data, we would like to infer the parameters *F*(*ω*) and *D*(*ω*) of Eq (1).

**Fig 2:**
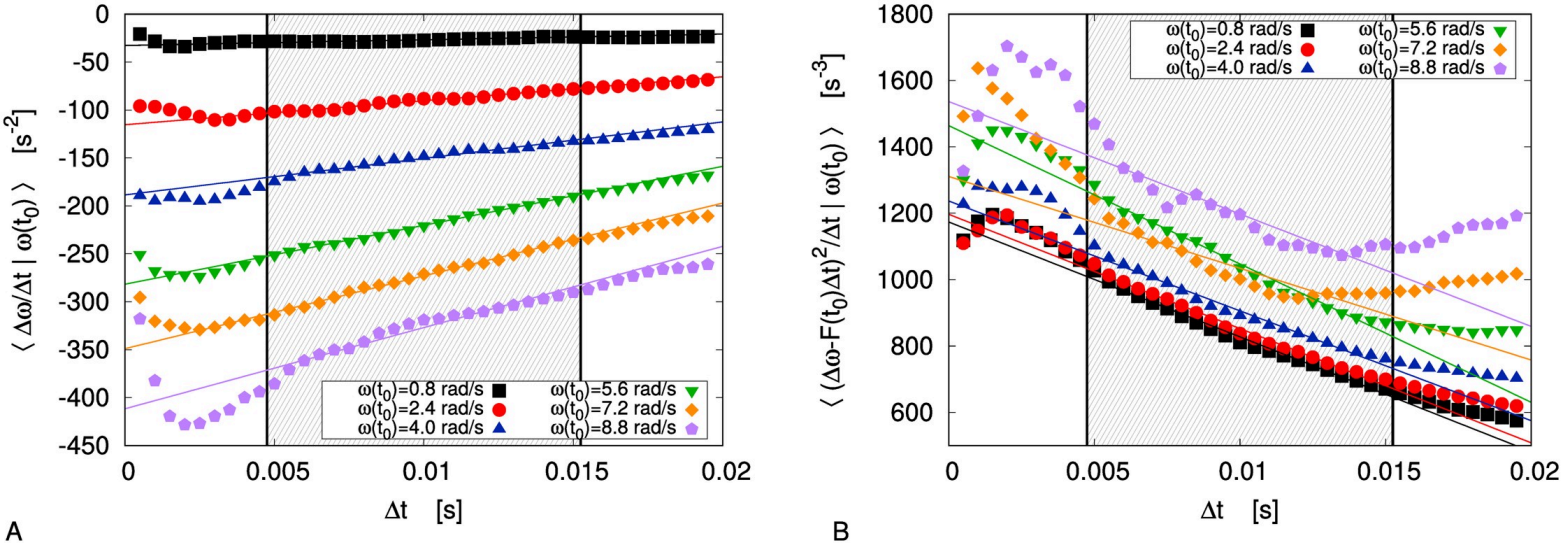
Gas limit: Extrapolation. Extrapolation of the limits on the r.h.s. of Eq (2), for several values of *ω*(*t*_0_), in order to compute drift (panel A) and diffusivity (panel B) in the gas limit. Each linear fit (solid lines) has been computed—by means of a classical least-square method—considering only the data in the shaded range, within the vertical lines.

In Fig 2 we plot the average quantities that appear on the r.h.s. of Eq (2), for several values of the time interval Δ*t*. As discussed in [8] and recalled in the previous section, when studying data series resulting from deterministic physical processes, the Markovian approximation can be considered true only at suitable time scales, namely for *τ*_*ME*_ ≪ Δ*t* ≪ *τ*, where *τ*_*ME*_ is the Markov-Einstein time and *τ* is a characteristic time for the autocorrelation function of the considered process. As a consequence, the limits on the r.h.s. of Eq (2) should be evaluated as extrapolations of the trend presented by data in a suitable time-scale range (Δ*t* ∈ [0.005, 0.015]*s* in our case, shaded region in Fig 2). We can perform a linear extrapolation using the least-square method: the vertical intercept of the resulting graph is our guess for the limit Δ*t* → 0. In order to evaluate a confidence interval for such value, one could estimate the uncertainty of each point of the graph and then consider the error propagation on the vertical intercept; however, since the data are not independent, this method is expected to underestimate the uncertainty. A safer way to compute the confidence interval is the “jackknife method” [45]: here we have divided our sampled data into *n* = 100 blocks, then we have repeated the analysis *n* times, discarding one block at each turn, and we have computed the confidence interval from the distribution of the resulting *n* different expected values.

**Fig 3:**
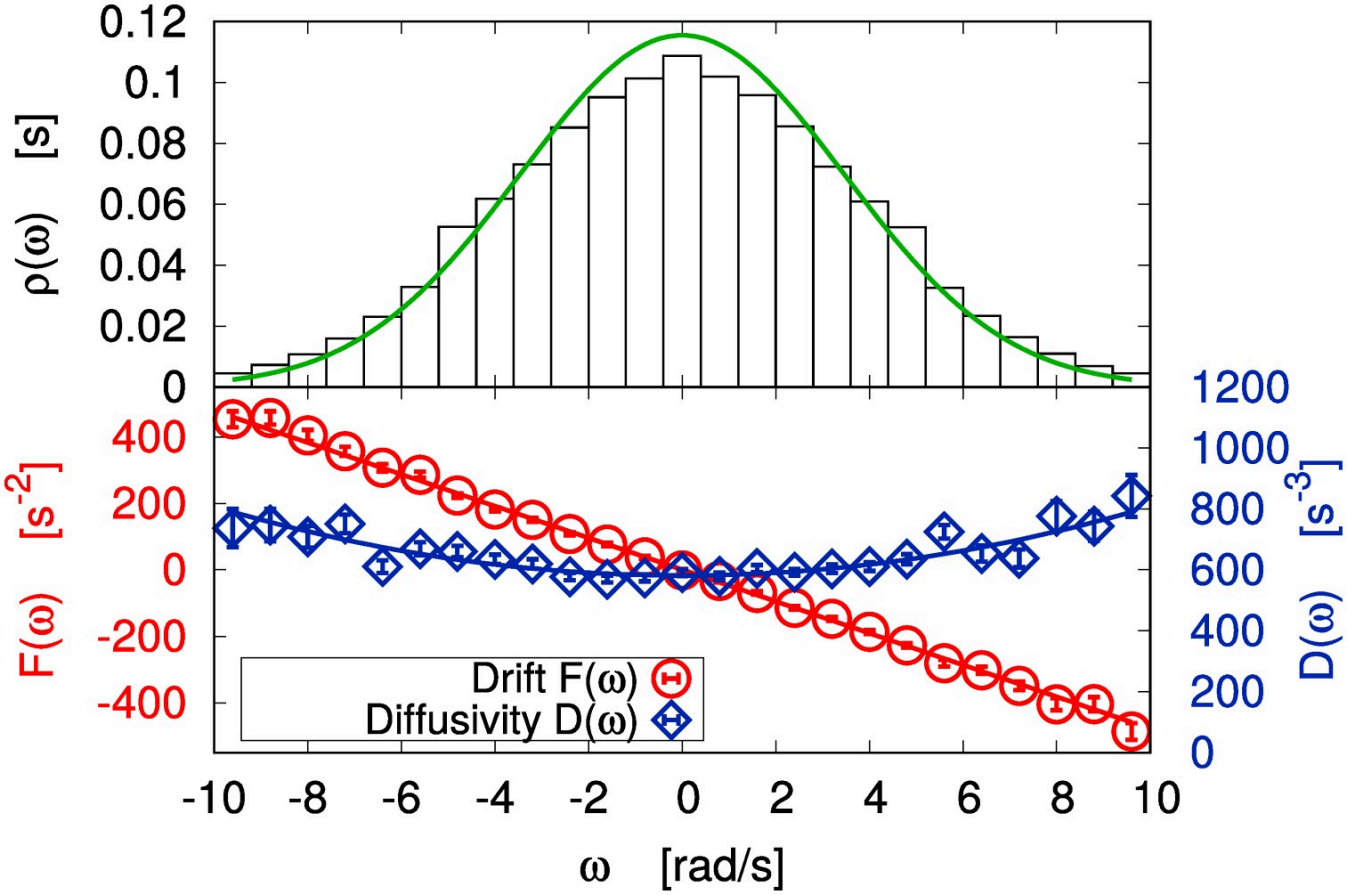
Gas limit: PDF, drift and diffusivity. Top: Probability distribution (PDF) of *ω* in the gas limit (the green solid line is the prediction of the derived model). Bottom: Reconstructed drift (red circles) and diffusivity (blue diamonds) in the gas limit have been respectively fitted with a linear and a parabolic function.

Taking the Δ*t* → 0 limit of the extrapolated linear trends, we have an estimate for the drift coefficient *F*(*ω*) and for the diffusivity *D*(*ω*): as it is shown in Fig 3 (bottom), the former has a linear dependence *F*(*ω*) = −*Aω* on the angular velocity, while the latter can be approximated as *D*(*ω*) = *D*_1_ + *D*_2_*ω*^2^. Of course, our procedure gives more accurate results when the angular velocity is smaller, i.e. when a bigger volume of data is available for the averages (see Fig 3 (top)). Let us notice that the quadratic corrections to the diffusivity are only relevant when |*ω*| is quite large, i.e. when our estimate is less reliable because of the low volume of data. For this reason, in the following we neglect such corrections and apply the constant approximation *D*(*ω*) = *D*. Model (1) reduces then to the well known Ornstein-Uhlenbeck process [4], so that all interesting physical observables can be computed analytically.

**Fig 4:**
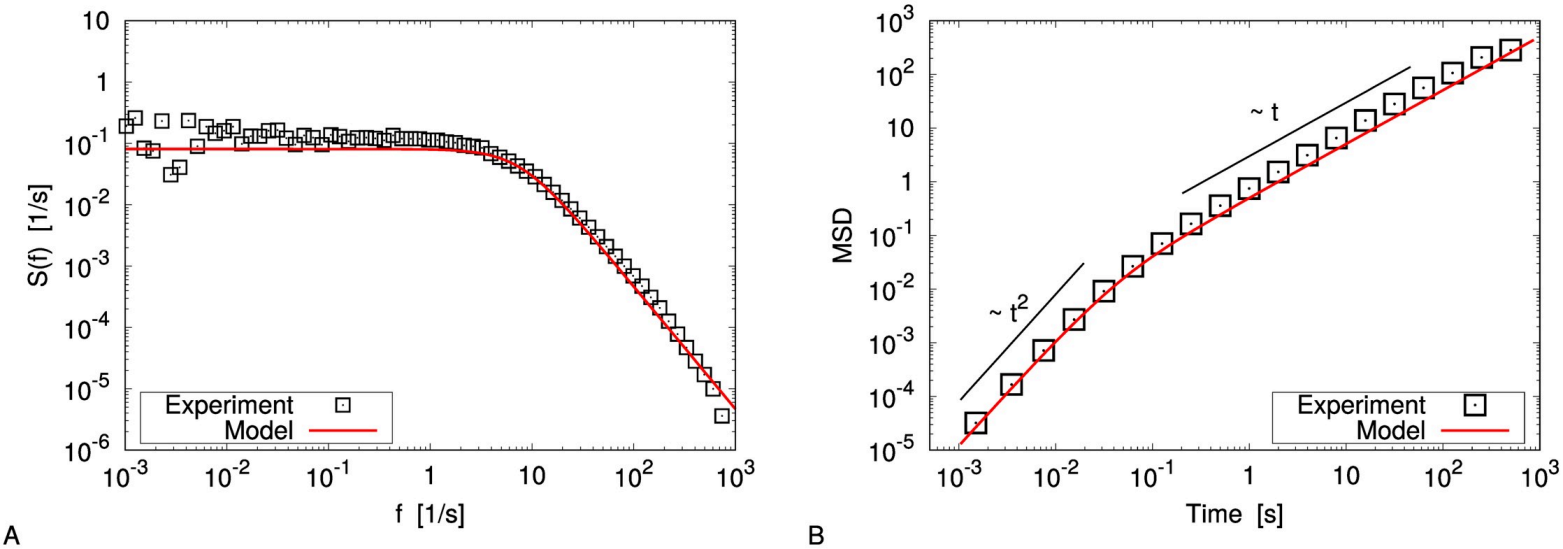
Gas limit: Observables. Velocity power density spectrum (panel A) and mean square displacement (panel B) in the gas limit. Experimental data (black squares) are compared with the reconstructed model (red lines). Black lines are guides for the eyes.

In Fig 3 (top) we observe a fair agreement between the predicted stationary probability distribution of *ω* and the experimental one. Table 1 summarizes the expected values for the parameters of the model, and the corresponding uncertainties. In Fig 4 we compare the experimental VPDS and MSD with the theoretical ones for the reconstructed Ornstein-Uhlenbeck process, finding a good agreement. The gas limit can be fairly approximated by this model, as already discussed in [25]. It is useful to recall that the characteristic time of the Ornstein-Uhlenbeck process (i.e. the decay of the velocity autocorrelation) is proportional to the mean free time between particle-blade collisions and in certain conditions can be quantitatively predicted [46]. We stress that if one considers also the quadratic corrections and performs numerical simulations, the outcomes are almost identical, at least in this case.

**Table 1:** Gas limit: Parameters. Expected values and uncertainties for the parameters of the reconstructed model in the gas limit.

Parameter	Value
*F*	(47.82 ± 0.42)*s*^−1^
*D*	(581.6 ± 5.8)*s*^−3^

### Cold liquid limit

In the following we analyze a regime which is somehow “opposite” to the gas limit seen above: in this case we consider *N* = 2600 beads and a shaking intensity Γ = 39.8; the packing fraction is *ϕ* = 36%. Again, *f* = 2000*Hz* and the experiment has a duration of 1 hour.

As already understood in [25, 43], in this case the rich phenomenology of the system cannot be described by a single-variable approach, since the dynamics of the granular matter involves at least two clearly separate time scales. Before enforcing the extrapolation procedure, we should be able to identify a “fast” variable and a “slow” one in order to understand how the model depends on them.

A quite straightforward way to define a variable that describes the slow behavior of the probe is to consider a running average with a Gaussian window function:

$$\begin{array}{c} \theta_{0}\left(t\right)=\frac{1}{\sqrt{2\pi }\sigma }\int dt^{\prime }\;e^{-\frac{{\left(t^{\prime }-t\right)}^{2}}{\sigma^{2}}}\theta \left(t^{\prime }-t\right)\;. \end{array}$$ (7)

**Fig 5:**
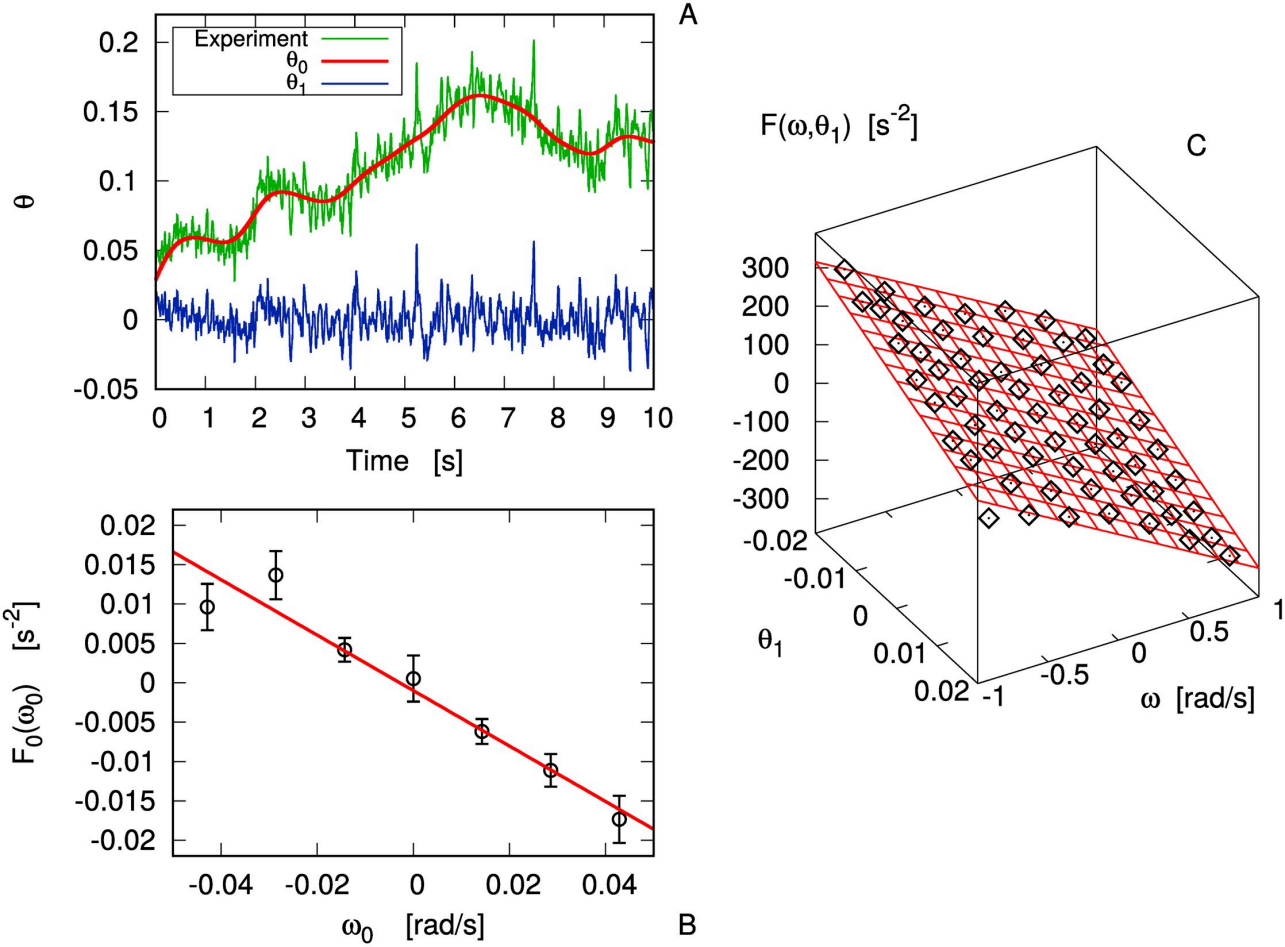
Cold liquid: Slow and fast components. A: Decomposing the original signal (green line) into the sum of the averaged angular position *θ*_0_ defined by Eq (7) (red line) and the “fast” variable *θ*_1_ = *θ* − *θ*_0_ (blue line). B: Drift coefficient of *ω*_0_. C: Drift coefficient of *ω*, as a function of *θ*_1_ and *ω*_0_.

The fast component can be found, of course, as *θ*_1_(*t*) = *θ*(*t*) − *θ*_0_(*t*) (see Fig 5A). The value of the characteristic time *σ* (here *σ* = 0.3*s*) is suggested by the shape of *S*(*f*), see Fig 1: we need to filter out the features in regions III and IV, but we also demand that the interesting dynamics in region I is reproduced by the new variable; taking 2*σ* ≃ *O*(1)*s* seems therefore a legitimate choice. In the following we will show that varying the parameter does not affect the results of our analysis significatively, as long as *σ* is chosen to be of the same order of magnitude. Of course, any other choice for the kernel in Eq (7) could be made, provided that it canceled the fast oscillations of *θ*(*t*) (i.e. provided that it had a Fourier transform decaying fast enough).

Let us notice that, following the physical interpretation proposed in [43], *θ*_0_ can be seen as the center of mass of the itinerant “cage” at every time, while *θ*_1_ has the meaning of the angular distance between *θ*_0_ and the probe itself.

First, we can use the extrapolation analysis seen before in order to determine a proper Langevin equation for the slow variable $\omega_{0}={\dot{\theta }}_{0}$. In this case the significative Δ*t* range can be found at a much slower time scale (namely, Δ*t* ∈ [1.5, 6]*s*): as a consequence, the volume of available data considerably shrinks, but it is still possible to estimate the dependence of the drift term on the slow angular velocity. In particular one finds (Fig 5B) that *F*(*ω*_0_) = −*A*_0_*ω*_0_ is an acceptable approximation. As in the previous case, we approximate the diffusivity term with a constant, *D*_0_(*ω*) = *B*_0_, neglecting the deviations for large |*ω*_0_|.

We are left with the problem of finding a model for the observed variable *ω*(*t*). In Fig 5C we show that the drift coefficient of *ω* depends significantly not only on *ω* itself, but also on *θ*_1_ = *θ* − *θ*_0_. A linear function of both arguments, *F*(*ω*, *θ*_1_) = −*A*_1_*ω* − *A*_2_*θ*_1_, turns out to provide a fair description of the data. Again we consider a constant value for the diffusivity, *D*(*θ*_1_, *ω*) = *B*.

The reconstructed model reads as follows:

$$\begin{array}{c} \begin{array}{cc} \dot{\omega }\left(t\right) & =-A_{1}\omega \left(t\right)-A_{2}\left[\theta \left(t\right)-\theta_{0}\left(t\right)\right]+\sqrt{2B}\eta \left(t\right)\\ \dot{\omega_{0}}\left(t\right) & =-A_{0}\omega_{0}\left(t\right)+\sqrt{2B_{0}}\eta_{0}\left(t\right)\\ \dot{\theta }\left(t\right) & =\omega (t)\\ {\dot{\theta }}_{0}\left(t\right) & =\omega_{0}\left(t\right) \end{array} \end{array}$$ (8)

where *η*(*t*) and *η*_0_(*t*) are Gaussian noises with unitary variance. Such system is a particular limit of the model guessed in [43], where the term *k*[*θ*(*t*) − *θ*_0_(*t*)] is negligible with respect to the other terms in Eq (6): indeed with the present definition, *ω*_0_ evolves with a slow dynamics that does not admit fluctuations on the fast time scale of *θ*_1_, so that such term is necessarily negligible.

**Fig 6:**
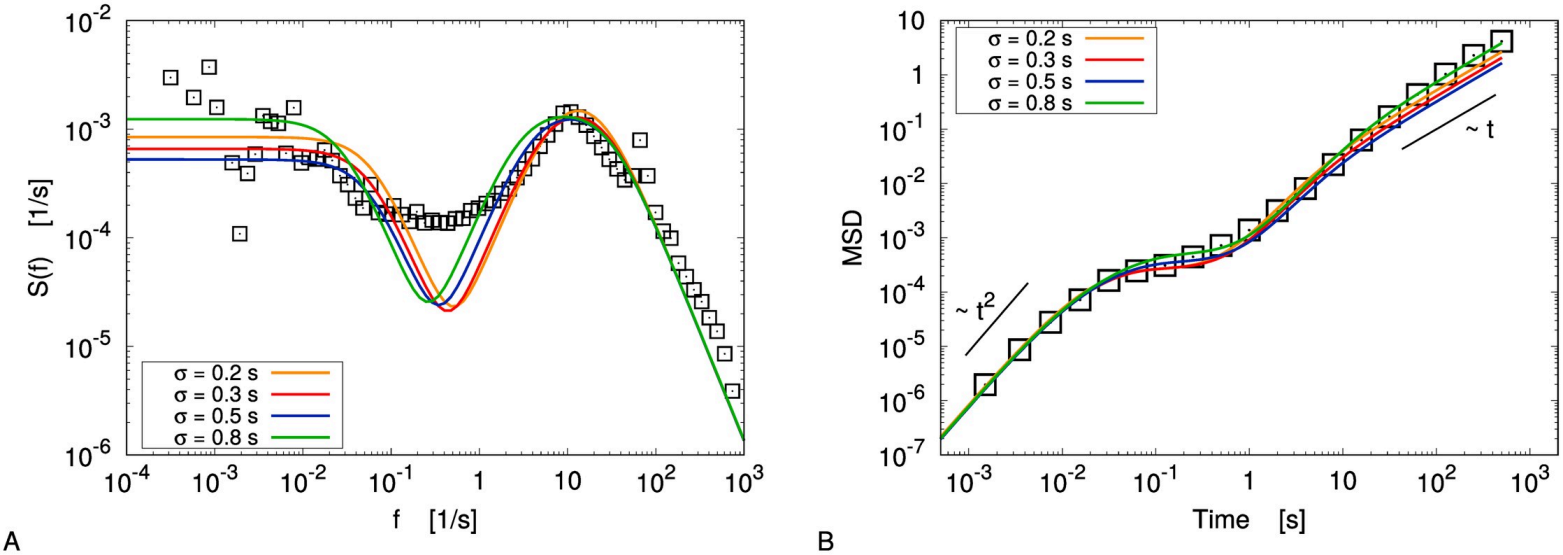
Cold liquid: Observables. Velocity power density spectrum (panel A) and mean square displacement (panel B) in the cold liquid case with Γ = 39.8. Experimental data (black squares) are compared with the reconstructed models (coloured lines) for several values of the smoothing parameter *σ*. Black lines are guides for the eyes.

The power density spectrum of *ω*(*t*) for model (8) can be determined analytically:

$$\begin{array}{c} S\left(\hat{f}\right)=\frac{1}{\pi }\frac{A_{2}^{2}B_{0}+A_{0}^{2}B{\hat{f}}^{2}+B{\hat{f}}^{4}}{A_{0}^{2}A_{2}^{2}+\left[A_{2}^{2}+A_{0}^{2}A_{1}^{2}-2A_{0}^{2}A_{2}^{2}\right]{\hat{f}}^{2}+\left[A_{1}^{2}-2A_{2}+A_{0}^{2}\right]{\hat{f}}^{4}+{\hat{f}}^{6}} \end{array}$$ (9)

where $\hat{f}=2\pi f$. Once $S(\hat{f})$ is known, the MSD can be found as

$$\begin{array}{c} \langle {\left[\Delta \theta \left(t\right)\right]}^{2}\rangle =\int_{0}^{t}dt^{\prime }\int_{0}^{t}dt^{\prime \prime }\langle \omega \left(t^{\prime }\right)\omega \left(t\right)\rangle =2\int_{0}^{t}dt^{\prime }\left(t-t^{\prime }\right)C_{\omega \omega }\left(t^{\prime }\right) \end{array}$$ (10)

where *C*_*ωω*_(*t*), the autocorrelation function of *ω*(*t*), is the Fourier anti-transform of $S(\hat{f})$. In Fig 6 we compare the above analytical expressions to the experimental data.

The VPDS shows a fair agreement in the high-frequency regime, *f* ≫ 1*Hz*, and in the low-frequency one *f* ≪ 1*Hz*; in the intermediate range (region II in the notation of [25]) there is a clear discrepancy between the model and the experimental data, maybe due to decorrelations of the slow variable that are not caught by the model. However, we stress that the difference concerns a frequency range which is almost inessential for the dynamical properties of the system, whose characteristic frequencies lay in regions I and III of the spectrum (see Fig 1B): this is completely evident when considering the MSD evolution (Fig 6B), that is very well reproduced by the model despite the discrepancy on $S(\hat{f})$. Let us note that changing *σ* by a factor 4, from 0.2*s* to 0.8*s*, does not affect the results of our analysis in a significative way.

Finally, let us consider an experiment with *N* = 2600, Γ = 26.8, *ϕ* = 45%: even if the number of beads is the same as in the previous case, the lower shaking intensity entails that the accessible volume for the beads is lower, i.e. the actual packing fraction increases. The system is therefore in a “more concentrate” state. The duration of the experiment has also been raised (12 hours), and we have chosen *σ* = 0.3*s* for the analysis.

**Fig 7:**
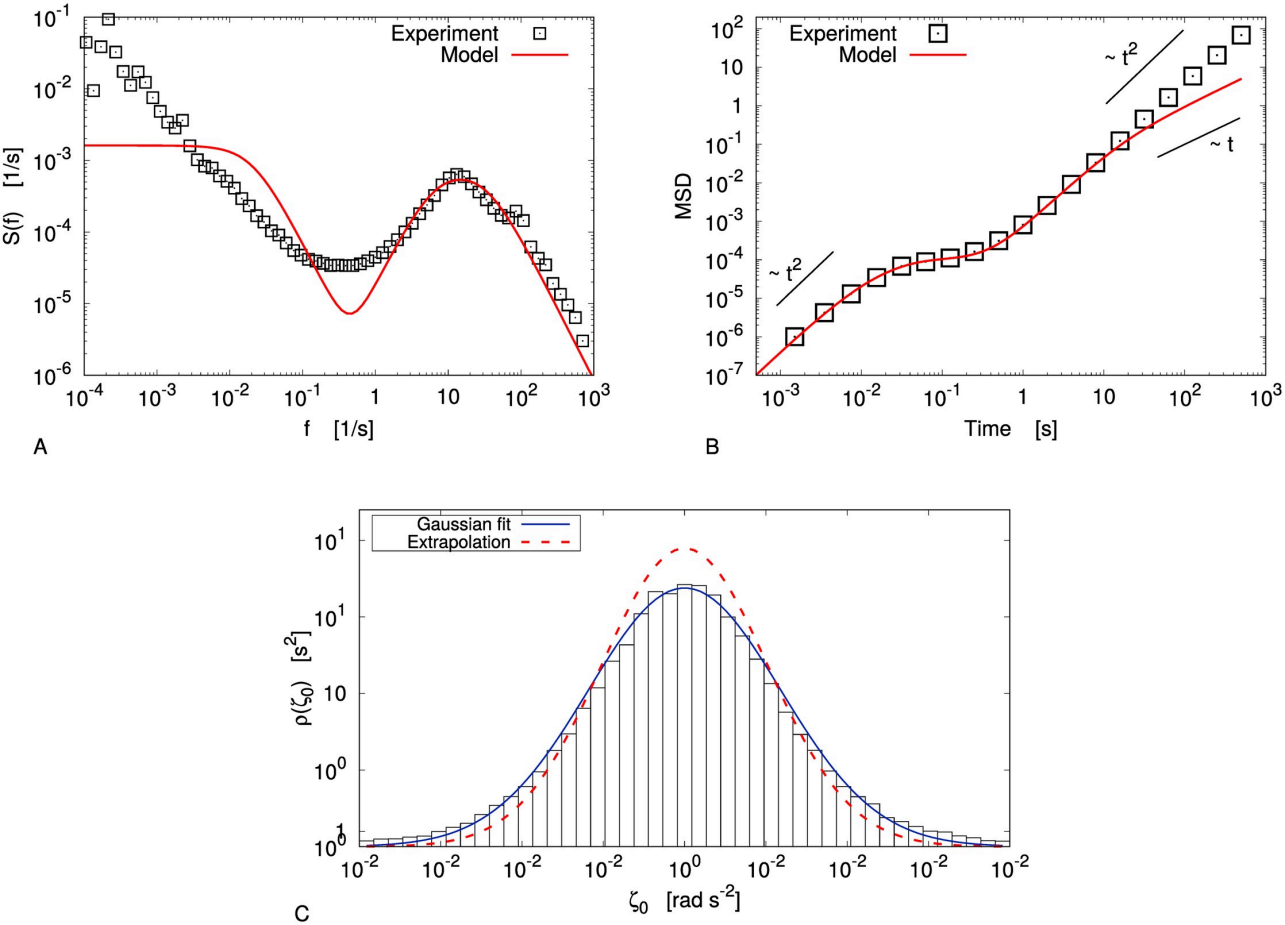
Cold liquid: Limits of the method. A: Velocity power density spectrum in the cold liquid limit with Γ = 26.8. B: Mean square displacement in the same case. C: Distribution of the “noise” *ζ*_0_ defined by Eq (11) (with Δ_*t*_ = 0.6*s*), Gaussian fit (blue line) and comparison with the reconstructed model (dashed red line).

Fig 7A and 7B show the VPDS and the MSD in this case, compared to those that can be inferred through the method discussed above. Even if the high-frequency regime is well reproduced by the model, our description fails on the long-time scale; in particular, the MSD of the reconstructed model shows a linear dependence on time for *t* ≳ 50*s*, while the experimental one is still quadratic on that scale.

The failure of the method could be ascribed to the choice of Gaussian noise for the slow variable: if the evolution of *ω*_0_ was ruled by a Lévy process, an alternative analysis should be considered [47]. Let us evaluate, *a posteriori*, the noise of the slow variable as:

$$\begin{array}{c} \zeta_{0}\left(t\right)=\frac{\omega_{0}\left(t+\Delta t\right)-\omega_{0}\left(t\right)}{\Delta t}-F_{0}\left(\omega_{0}\left(t\right)\right)\;. \end{array}$$ (11)

Fig 7C shows that the distribution of *ζ*_0_ can be fairly approximated with a Gaussian; furthermore, the amplitude of the noise is very close to that of the reconstructed model. Hence, we can guess that the hypothesis of Gaussian noise is quite reasonable, and the discrepancy between the experimental data and the inferred model could need a different explanation.

Table 2 summarizes the expected values for the parameters of model (8), and the corresponding uncertainties, for the two considered cases in the cold liquid limit. Also in this case the confidence intervals have been computed using the jackknife method on *n* blocks of sampled data: we have chosen *n* = 100 for the fast variables, *n* = 10 for the slow ones.

**Table 2:** Cold liquid: Parameters. Expected values and uncertainties for the parameters of model (8) in the cold liquid limit.

Parameter	Γ = 39.8	Γ = 26.8
*A*_1_ [*s*^−1^]	200.3 ± 4.4	252.0 ± 6.3
*A*_2_ [*s*^−2^]	5.76 ⋅ 10^3^ ± 2.4 ⋅ 10^2^	8.55 ⋅ 10^3^ ± 4.5 ⋅ 10^2^
*B* [*s*^−3^]	161.1 ± 3.9	107.3 ± 3.1
*A*_0_ [*s*^−1^]	0.352 ± 0.034	0.1317 ± 4.9 ⋅ 10^−3^
*B*_0_ [*s*^−3^]	2.43 ⋅ 10^−4^ ± 2.1 ⋅ 10^−5^	8.82 ⋅ 10^−5^ ± 2.7 ⋅ 10^−6^

## Conclusion

Using data from an accurate experiment on the rotational diffusion in granular media we derived a Langevin equation which is able to describe the main dynamical statistical features of the system.

Let us stress that, in the building of the model, part of the protocol is quite standard. On the other hand there are rather subtle conceptual aspects which cannot be ignored. In the dilute case it is enough to use a single variable whose dynamics is well described by a linear Langevin equation. Much more difficult is the dense case where it is not obvious, as already stressed in the past by many eminent scientists [14, 15], the set of variables which is ruled by a Markov process.

At a first glance, it seems natural to follow the approach of Takens for the phase space reconstruction [10]. The basic idea is: from the knowledge of a time series {*u*(*t*)} one starts with the variable {*u*(*t*)} itself, if this choice is not appropriate one can try with {*u*(*t*), *du*(*t*)/*dt*}, then {*u*(*t*), *du*(*t*)/*dt*, *d*^2^*u*(*t*)/*dt*^2^}, and so on. Once the dimension of the phase space has been fixed one can try to find the evolution equation, certainly with many practical problems to solve, see [48]. In our system one faces an additional important obstacle. Consider a series {*u*(*t*)} with a characteristic time *τ*_*c*_, surely the variables *du*(*t*)/*dt*, *d*^2^*u*(*t*)/*dt*^2^ etc. have characteristic times which cannot be larger that *τ*_*c*_, therefore a protocol based on the Takens’ approach cannot succeed in systems with a multiscale structure, i.e. with other relevant variables whose time scales are much longer than *τ*_*c*_.

Actually in our case in order to find a good model we have been forced to go in a direction which is the opposite of the Takens method; i.e. to identify a slow variable obtained with a convolution, which is somehow antithetical to a derivative.

## Supporting information

S1 File Experiment with *N* = 350, Γ = 39.8. Angular position *θ* of the blade during the experiment with 350 spheres, maximum acceleration 39.8*g* and duration of 1 hour. Sampling rate of 2000*Hz*.
(ZIP)

S2 File Experiment with *N* = 2600, Γ = 39.8. Angular position *θ* of the blade during the experiment with 2600 spheres, maximum acceleration 39.8*g* and duration of 1 hour. Sampling rate of 2000*Hz*.
(ZIP)

S3 File Experiment with *N* = 2600, Γ = 26.8: First part. Angular position *θ* of the blade during the experiment with 2600 spheres, maximum acceleration 26.8*g* and duration of 12 hours. Sampling rate of 2000*Hz*.
(ZIP)

S4 File Experiment with *N* = 2600, Γ = 26.8: Second part. Angular position *θ* of the blade during the experiment with 2600 spheres, maximum acceleration 26.8*g* and duration of 12 hours. Sampling rate of 2000*Hz*.
(ZIP)
